# Up-regulated circBACH2 contributes to cell proliferation, invasion, and migration of triple-negative breast cancer

**DOI:** 10.1038/s41419-021-03684-x

**Published:** 2021-04-19

**Authors:** Xinxing Wang, Bingjian Xue, Yujie Zhang, Guangcheng Guo, Xin Duan, Dongwei Dou

**Affiliations:** Department of Breast Surgery, the First Affiliated Hospital of Zhengzhou University, No.1 Jianshedong Road, Erqi District, Zhengzhou, Henan Province People’s Republic of China

**Keywords:** Cancer, Cell biology

## Abstract

An increasing amount of evidence has proven the vital role of circular RNAs (circRNAs) in cancer progression. However, there remains a dearth of knowledge on the function of circRNAs in triple-negative breast cancer (TNBC). Utilizing a circRNA microarray dataset, four circRNAs were identified to be abnormally expressed in TNBC. Among them, circBACH2 was most significantly elevated in TNBC cancerous tissues and its high expression was positively correlated to the malignant progression of TNBC patients. In normal human mammary gland cell line, the overexpression of circBACH2 facilitated epithelial to mesenchymal transition and cell proliferation. In TNBC cell lines, circBACH2 knockdown suppressed the malignant progression of TNBC cells. Mechanistically, circBACH2 sponged miR-186-5p and miR-548c-3p, thus releasing the C-X-C chemokine receptor type 4 (CXCR4) expression. The interference of miR-186-5p/miR-548c-3p efficiently promoted the cell proliferation, migration, and invasion suppressed by circBACH2 knockdown in the TNBC cell lines. Finally, circBACH2 knockdown repressed the growth and lung metastasis of TNBC xenografts in nude mice. In summary, circBACH2 functions as an oncogenic circRNA in TNBC through a novel miR-186-5p/miR-548c-3p/CXCR4 axis.

## Introduction

Triple-negative breast cancer (TNBC) accounts for ~15% of breast cancers and is characterized by high proliferation and frequent metastasis to the lungs and the brain^1,2^. Due to the lack of estrogen receptor (ER), progesterone receptor, and human epidermal receptor 2 (HER2), TNBC patients are unable to benefit from general hormonal or HER2-targeted therapies and usually have awful prognoses^3^. According to statistics, about 30–40% of TNBC patients die from the recurrence and metastasis of TNBC^4^. To date, chemotherapeutics and radiation therapy are still the standard treatments for TNBC patients^5^. Therefore, there is an urgent need to elucidate the underlying mechanisms of TNBC progression and metastasis, so as to provide new therapeutic interventions for the treatment of TNBC patients.

Circular RNA (circRNA) is a type of non-coding RNA that is characterized by covalently closed-loop structures; it is highly conserved in mammalian cells^6^. At present, the potential roles of circRNAs in breast cancer are becoming a novel focus of research. An increasing number of researchers have revealed that circRNAs participate in breast cancer progression by acting as the sponges of microRNAs (miRNAs), binding to the miRNA response elements directly and weakening their ability to combine with target mRNA. For instance, circKDM4C releases phenazine biosynthesis-like protein domain-containing protein expression by sponging miR-548p, thus attenuating doxorubicin resistance in breast cancer^7^. circBMPR2 functions as a tumor suppressor gene in breast cancer through the miR-533/ubiquitin-specific protease 4 axis^8^. In addition, the potential role of circRNAs in TNBC is gradually being revealed. He et al^9^. find that circGFRA1 is overexpressed in TNBC and its high expression was correlated with the poor overall survival of TNBC patients. Zeng et al^10^. define circANKS1B as a biomarker for the lymphatic metastasis of TNBC. However, to date, the functions of the majority of circRNAs in TNBC remain unclear.

In the present study, we investigated abnormally expressed circRNAs in TNBC using a microarray dataset (GSE101123) and clinical samples of TNBC cancerous tissues. We identified a novel TNBC-related circRNA hsa_circ_000442, which is derived from the BACH2 gene locus, called circBACH2. Subsequently, we explored the function and underlying molecular mechanism of circBACH2 in TNBC progression and metastasis in vitro and in vivo, hoping to further elucidate the crucial role of circRNAs in TNBC and provide new perspectives for the development of clinical therapeutic strategies against TNBC.

## Materials and methods

### Patient samples

A total of 38 patients who were diagnosed with TNBC at the First Affiliated Hospital of Zhengzhou University were enrolled in this study. The 38 pairs of TNBC cancerous tissues and adjacent normal tissues (ANT) were obtained through surgical resection, after which they were immediately preserved in RNAlater (Qiagen, Germany) and stored at −80 °C. This study was approved by the Ethics Committee of the First Affiliated Hospital of Zhengzhou University and all patients signed informed consent forms. The clinical-pathological features of TNBC patients we enrolled in are shown in Table 1.

**Table 1:** Relationship between circBACH2 expression and clinical-pathological features of TNBC patients (*n* = 38).

Variables	Cases (*n* = 38)	circBACH2		*P* value
		Low (*n* = 19)	High (*n* = 19)	
Age (years)				
<50	9	7 (77.8%)	2 (22.2%)	0.125
≥50	29	12 (41.4%)	17 (58.6%)	
Menopause				
No	15	6 (40.0%)	9 (60.0%)	0.508
Yes	23	13 (56.5%)	10 (43.5%)	
T stage				
T1–T2	16	12 (75.0%)	4 (25.0%)	0.020
T3–T4	22	7 (31.8%)	15 (68.1%)	
N stage				
N0	18	14 (77.8%)	4 (22.2%)	0.003
N1–3	20	5 (25.0%)	15 (75.0%)	
TNM stage				
I–II	17	15 (88.2%)	2 (11.8%)	<0.001
III–IV	21	4 (19.1%)	17 (81.0%)	

*TNBC* triple-negative breast cancer, *TNM* tumor-node-metastasis.

A value of *P* < 0.05 was considered statistically significant.

### Cell culture and transfection

The TNBC cell lines (MDA-MB-231, MDA-MB-468, MDA-MB-453, and BT-549), normal mammary gland cell line MCF-10A, ER^+^/HER2^−^ cell line MCF-7^11^, and ER^+^/HER2^+^ cell line BT-474^11^ were commercially obtained from the Procell Life Science&Technology Co., Ltd (China). MDA-MB-231, MDA-MB-468, and MDA-MB-453 were maintained in Leibovitz’s L-15 medium supplemented with 10% FBS at 37 °C. BT-549 were maintained in RPMI-1640 medium supplemented with 10% FBS (Solarbio, China) in 5% CO_2_ ambiance at 37 °C.

For circBACH2 overexpression, pLCDH-ciR vector (Geneseed, China) containing the cDNA of circBACH2 was employed (named as circBACH2). Mock vector was used as a negative control (named as vector). For circBACH2 silencing, pLL3.7 vector (FENGHUISHENGWU, China) containing the shRNA against circBACH2 was employed (named as sh-circBACH2). ShRNA-NC was served as a negative control (named as sh-NC). miR-186-5p/miR-548c-3p inhibitor and miR-186-5p/miR-548c-3p mimic were provided by RiboBio (China) commercially. Cell transfection was implemented with the assistance of Lipofectamine 3000 (Thermo Fisher, USA). Forty-eight hours later, the transfection efficiency was confirmed through qRT-PCR.

### Cell cycle analysis

Before analysis, MCF-10A, MDA-MB-231, or BT-549 cells were transfected with vector/ circBACH2/sh-NC/sh-circBACH2. Then, well-grown cells were harvest and fixed using methanol. Cell Cycle Detection Kit (KeyGEN BioTECH, China) was employed to determine the cell cycle distribution.

### qRT-PCR

The RNAprep Pure Cell Kit and RNAprep Pure Tissue Kit (Tiangen, China) were used to extract the total RNA from the cells and tissues, respectively. Reverse transcription was conducted for the synthesis of cDNA using a PrimeScript RT Reagent Kit (Takara, Japan) or a miScript Reverse Transcription Kit (Qiagen, GER). PCR was conducted using the SYBR Premix Ex Taq II Kit (Takara) or a miScript SYBR Green PCR Kit (Qiagen, GER). The relative circBACH2, miR-186-5p, and miR-548c-3p expressions were calculated using the 2^˗ΔΔCT^ method. Table 2 listed the primer sequences used in this study. U6 or GAPDH was used as the endogenous control.

**Table 2:** Primer sequences for qRT-PCR.

Gene	Primer sequences
CircBACH2	F: 5′-TACCTGGAGGGTGGCATTTA -3′
	R: 5′- TTGCGTTTGAACAGTTCCTG-3′
CircCBFB	F: 5′-CGCTCGGAAATCGCTTTTGT-3′
	R: 5′-TCTCGGCTAGGTGTTTGTCG-3′
Circ-000638	F: 5′-CTATGTGCGGGCCATTGAGA-3′
	R: 5’-ATTGGAGATTCCGTGCTGCT-3’
CircUCK2	F: 5′-GCTCTCACGCAGAGTCTTCC-3′
	R: 5′-CCGAGGTAAGGACACGGTAG-3′
GAPDH	F: 5′-GAAGGTGAAGGTCGGAGTC-3′
	R: 5′-GAAGATGGTGATGGGATTTC-3′
miR-186-5p	F: 5′-CGCGCAAAGAATTCTCCTTT-3′
	R: 5′-AGTGCAGGGTCCGAGGTATT-3′
miR-548c-3p	F: 5′-GCGCGCAAAAATCTCAATTAC-3′
	R: 5′-AGTGCAGGGTCCGAGGTATT-3′
U6	F: 5′-CTCGCTTCGGCAGCACA-3′
	R: 5′-AACGCTTCACGAATTTGCGT-3′

### Colony formation assay

The 1000 MDA-MB-231 or BT-549 cells were seeded into six-well plates and cultured under normal conditions. Two weeks later, the plates were fixed with paraformaldehyde for 6 min. Then, the plates were stained with 0.1% crystal violet solution for 20 min at room temperature. The colonies with a diameter larger than 0.1 mm were counted.

### Invasion and migration assays

Before the cell invasion assay, a Transwell chamber was pre-coated with matrigel (BD Bioscience, USA). Then, the 100 µl of serum-free medium containing 1 × 10^5^ MDA-MB-231 or BT-549 cells was transferred to the upper chamber. A 500 µl of medium containing 15% FBS was added to the lower chamber. Twelve hours later, the cells that passed through the matrigel-coated filter were fixed with paraformaldehyde, followed by crystal violet (0.1%) staining. Finally, the stained cells were counted under a microscope. The experimental procedure of the migration assay was the same as that of the invasion assay, except that the chambers were not pre-coated.

### Dual-luciferase reporter assay

To validate the interplay between circBACH2 and miR-186-5p/miR-548c-3p, the sequence of circBACH2 wild type (WT) or mutant type (MUT) was amplified and inserted into the pmir-GLO vector (Promega, USA). Then, the vector containing circBACH2 WT/MUT and miR-186-5p mimic/miR-548c-3p mimic/mimic NC were co-transfected into well-grown MDA-MB-231/BT-549 cells using Lipofectamine 3000 (Thermo Fisher, USA). Two days later, the luciferase activities of circBACH2 WT and MUT were measured by a dual-luciferase reporter assay kit (Promega). To validate the interplay between miR-186-5p/miR-548c-3p and *CXCR4*, the luciferase activities of *CXCR4* 3′UTR WT and MUT were measured in the same way.

### RNA pull-down

The biotin-labeled circBACH2 probe and control probe were obtained from RiboBio (China) commercially. The lysates of the MDA-MB-231 or BT-549 cells were obtained using Pierce™ IP Lysis Buffer (Thermo Fisher) and incubated with the 3 µg biotin-labeled circBACH2 probe or control probe at 4 °C overnight. The mixture was incubated with 50 µl streptavidin magnetic beads (Invitrogen, USA) for 3 h at 4 °C. Subsequently, the complex bound by the biotin-labeled circBACH2 probe or control probe was eluted, followed by qRT-PCR.

### Fluorescence in situ hybridization (FISH)

To detect the subcellular localization of circBACH2 and miR-186-5p/miR-548c-3p, FISH was conducted. The Cy3-labeled circBACH2 probe and FAM-labeled miR-186-5p/miR-548c-3p probes were commercially obtained from RiboBio (China). The MDA-MB-231 and BT-549 cells growing on the coverslips were fixed with 4% paraformaldehyde for a quarter-hour. After treating with proteinase K, the slides were maintained for 30 min at 37 °C in the presence of a prehybridization solution (RiboBio, China). The slides were hybridized to the Cy3-labeled circBACH2 probe and FAM-labeled miR-186-5p/miR-548c-3p probes for 1 day at 42 °C. Then, the cell nucleus was counterstained using DAPI. The stained slides were visualized under a confocal microscope (Nikon, Japan).

### Western blot

After purifying from cell lysate or tissue homogenate, the protein samples were subjected to electrophoresis, followed by transferring to the PDVF membrane (Thermo Fisher Scientific, USA). After being blocked with 5% skim milk for 30 min, membranes were orderly incubated with primary antibodies, secondary antibodies, and ECL reagent. Then, immunoblots were visualized utilizing a Gel imager. The informations about primary antibodies were as follows: C-X-C chemokine receptor type 4 (CXCR4; 1:5000, Abcam, UK), E-cadherin (1 µg/ml, Abcam), occludin (1:1000, Abcam), Vimentin (1:3000, Abcam), Slug (2 µg/ml, Abcam), Twist (0.5 µg/ml, Abcam), Snail (1:1000, Abcam).

### In vivo xenograft assay

Lentivirus (Lv)-sh-circBACH2 and Lv-circBACH2, as well as their corresponding controls (Lv-sh-NC and Lv-NC) were commercially obtained from RiboBio (China). Female 6-week-old BALB/C nude mice (*n* = 48) were commercially obtained from Beijing Vital River Laboratory Animal Technologies Co. Ltd (China). All animal experiments were approved by the Ethics Committee of the First Affiliated Hospital of Zhengzhou University.

To evaluate the influence of circBACH2 on the growth of TNBC cells, the mice were divided into four groups: Lv-NC (*n* = 6), Lv-circBACH2 (*n* = 6), Lv-sh-NC (*n* = 6), and Lv-sh-circBACH2 (*n* = 6). A total of 5 × 10^6^ MDA-MB-231 cells were transfected with Lv-NC/Lv-circBACH2/Lv-sh-NC/Lv-sh-circBACH2 and then subcutaneously injected into the mice. Four weeks later, the mice were sacrificed and the tumor weight of each mouse was measured.

To observe the influence of circBACH2 on the lung metastasis of TNBC cells, the mice were divided into four groups: Lv-NC (*n* = 6), Lv-circBACH2 (*n* = 6), Lv-sh-NC (*n* = 6), and Lv-sh-circBACH2 (*n* = 6). A total of 2 × 10^6^ MDA-MB-231 cells were transfected with Lv-NC/Lv-circBACH2/Lv-sh-NC/Lv-sh-circBACH2 and then injected into the mice through the tail vein. Six weeks later, the mice were sacrificed and the lung tissues of each mouse were collected.

### Hematoxylin and eosin (HE) staining

The lung tissues of the mice were fixed with formaldehyde, embedded with paraffin, and then sectioned. The sections were orderly stained with hematoxylin and eosin for 9 and 4 min, respectively. The stained sections were then visualized under a microscope (Nikon, Japan).

### Statistical analysis

Data were representatives of three times replicates for the in vitro studies. Data were expressed as mean ± SD. Statistical analysis was performed using GraphPad Prism 6.0. The data from two experimental groups were analyzed by student’s *t*-test. The correlation between the circBACH2 level and the CXCR4 protein level/miR-186-5p level/miR-548c-3p level in TNBC cancerous tissues was analyzed by Pearson correlation analysis. Results were considered statistically significant when *P* < 0.05.

## Results

### circBACH2 was upregulated in TNBC cancerous tissues and TNBC cell lines

**Fig. 1:**
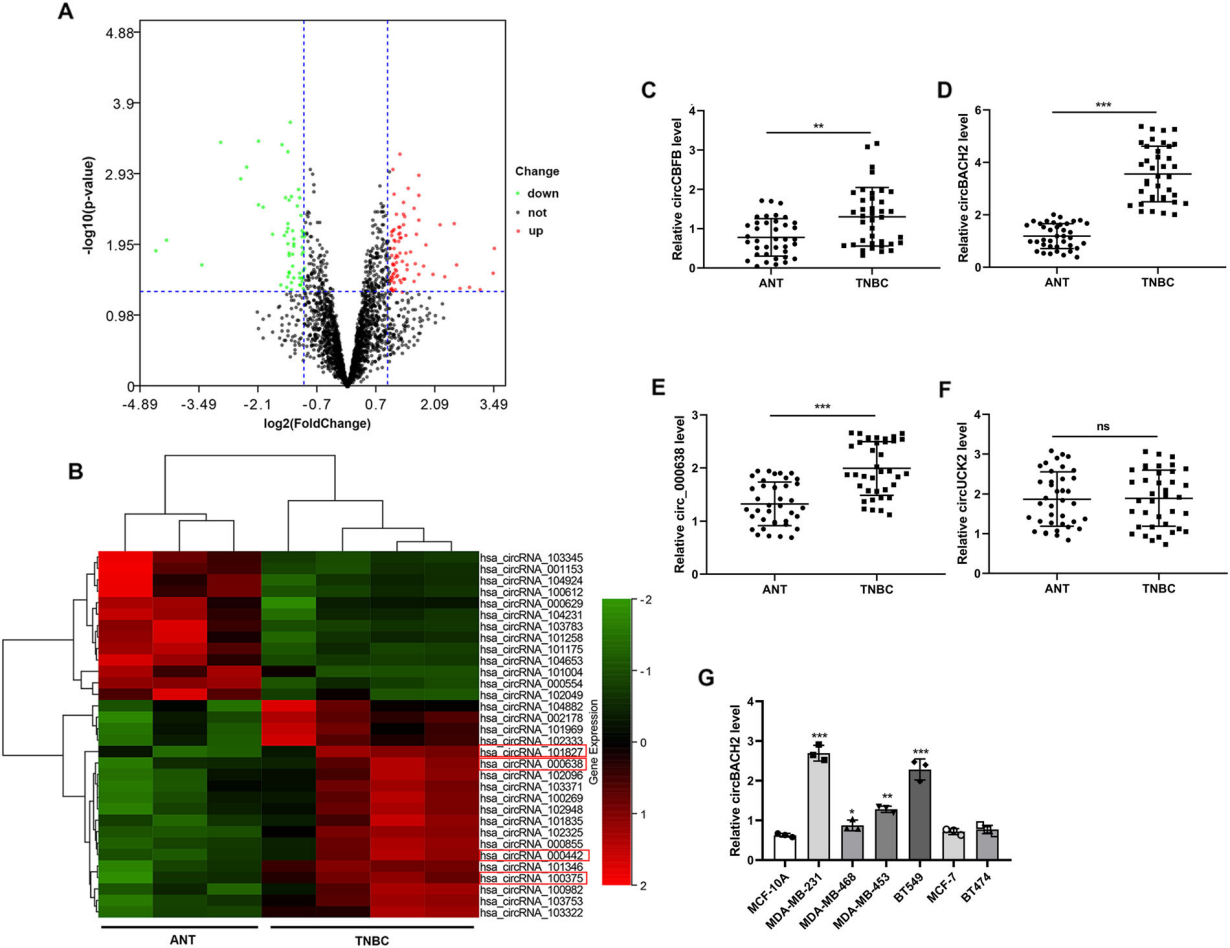
circBACH2 was upregulated in triple-negative breast cancer (TNBC) tissues and TNBC cell lines. The microarray dataset that is related to TNBC (GSE101123) was downloaded. **A** The volcano plot visualized the expressions of circRNA between TNBC cancerous tissues (*n* = 4) and adjacent normal tissues (ANT; *n* = 3). The red dots and green dots represent upregulated and downregulated circRNAs with statistical significance, respectively. **B** Clustered heat map of the differentially expressed circRNAs in TNBC cancerous tissues (*n* = 4) and ANT (*n* = 3). Rows represent circRNAs and columns represent tissue types. The color scale runs from green (low intensity) to black (medium intensity), to red (strong intensity). **C–F** Thirty-eight pairs of TNBC cancerous tissues and ANT were obtained from TNBC patients (*n* = 38). The expression levels of circCBFB, circ_000638, circBACH2, and circUCK2 were determined using qRT-PCR. **P < 0.01, ***P < 0.001. ns no significance. **G** The expression level of circBACH2 in the normal mammary gland cell line MCF-10A, TNBC cell lines (MDA-MB-231, MDA-MB-468, MDA-MB-453, and BT-549), estrogen receptor (ER)^+^/human epidermal receptor 2 (HER2)^−^ cell line MCF-7, and ER^+^/HER2^+^ cell line BT-474 was determined using qRT-PCR. *P < 0.05, **P < 0.01, ***P < 0.001 vs MCF-10A.

In order to evaluate the expressions of circRNAs in TNBC, we downloaded a microarray dataset that is related to TNBC (GSE101123) and analyzed using SangerBox. Volcano plot filtering revealed a variation of circRNA expressions between TNBC cancerous tissues and ANT (Fig. 1A). With the cut-off criteria of fold change ≥2 and *P* value <0.05, we found that 13 circRNAs were downregulated and 19 circRNAs were upregulated in TNBC cancerous tissues (Fig. 1B). Among these abnormally expressed circRNAs, the expression levels of four circRNAs that had been proven to be related to cancer progression [hsa_circ_101827 (circCBFB), hsa_circ_000638, hsa_circ_000442 (circBACH2), hsa_circ_100375 (circUCK2)]^12–15^ were measured in the clinical samples we collected. As depicted in Fig. 1C–F, circCBFB, circBACH2, and hsa_circ_000638 were highly expressed in the TNBC cancerous tissues compared with ANT. Among them, the expression level of circBACH2 demonstrated the most significant change. Similarly, the expression levels of circBACH2 in the TNBC cell lines, especially in MDA-MB-231 and BT-549, were much higher than in the normal mammary gland cell line (MCF-10A) and cell lines of other subtypes of breast cancer (MCF-7 and BT-474) (Fig. 1G). Meanwhile, as shown in Table 1, increased circBACH2 was statistically associated with the T stage, N stage, and TNM stage, indicating the vital role of circBACH2 in the progression of TNBC.

### circBACH2 enhanced in vitro migration-invasiveness of TNBC cell lines

**Fig. 2:**
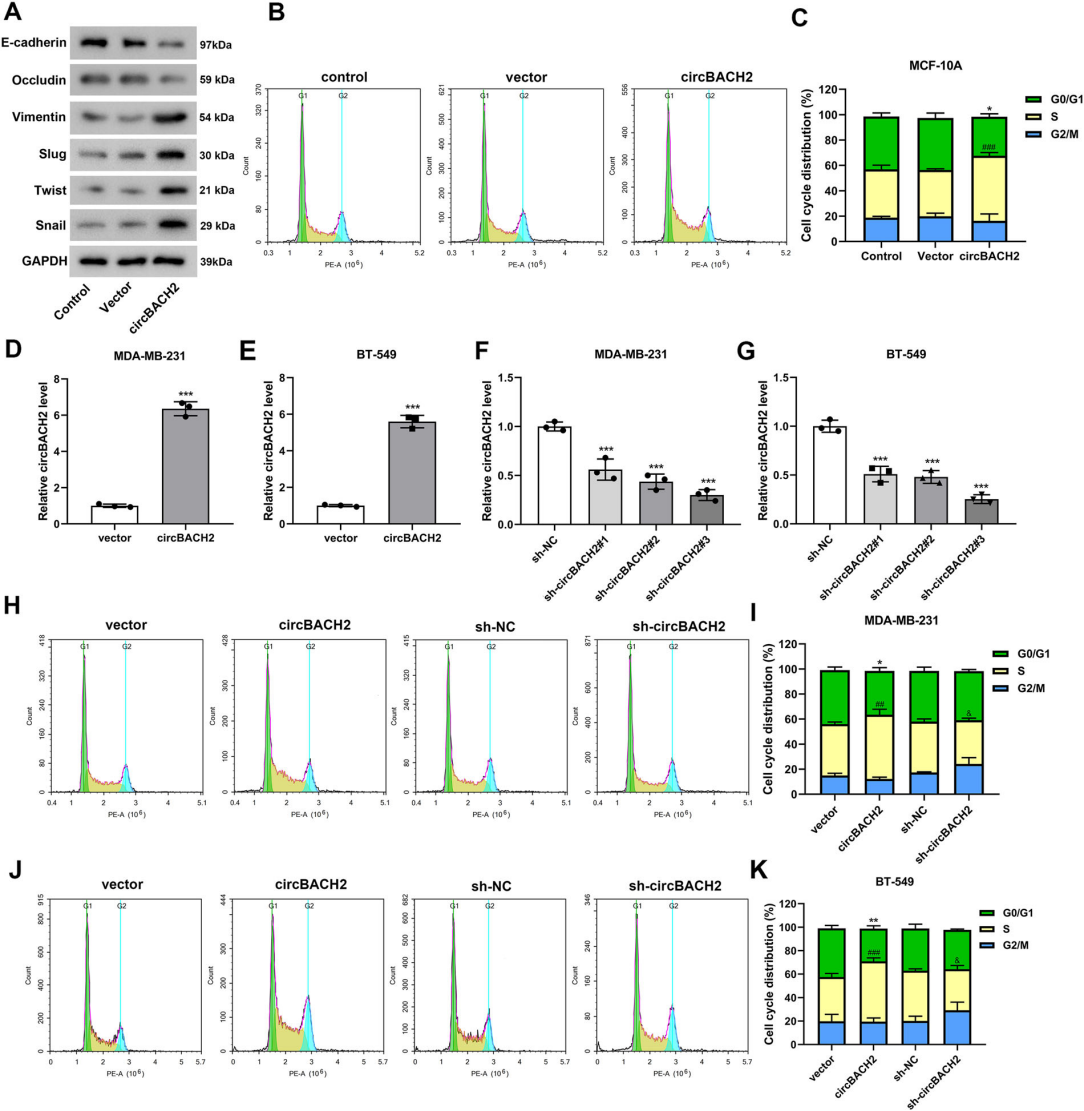
The oncogenic potential of circBACH2. **A**, **B** MCF-10A cells were transfected with the overexpression vector of circBACH2 (circBACH2) or mock vector (vector). **A** The protein levels of E-cadherin, Occludin, Vimentin, Slug, Twist, and Snail were measured by western blot. **B**, **C** Flow cytometry determined the relative cell numbers in each cell-cycle phase. *P < 0.05 vs G0/G1 phase of vector group; ^###^*P* < 0.001 vs S phase of vector group. **D**, **E** The transfection efficiency of the overexpression vector of circBACH2 was measured by qRT-PCR in TNBC cell lines. ***P < 0.001 vs vector. **F**, **G** The transfection efficiency of sh-BACH2#1, #2, and #3 was measured by qRT-PCR in TNBC cell lines. The sh-NC served as a negative control. ***P < 0.001 vs sh-NC. **H**–**K** Flow cytometry determined the relative cell numbers in each cell-cycle phase in the MDA-MB-231 and BT-549 cells that were transfected with indicated vectors. *P < 0.05, **P < 0.01 vs G0/G1 phase of vector group; ^##^*P* < 0.01, ^###^*P* < 0.001 vs S phase of vector group; and ^&^*P* < 0.05 vs S phase of sh-NC group.

**Fig. 3:**
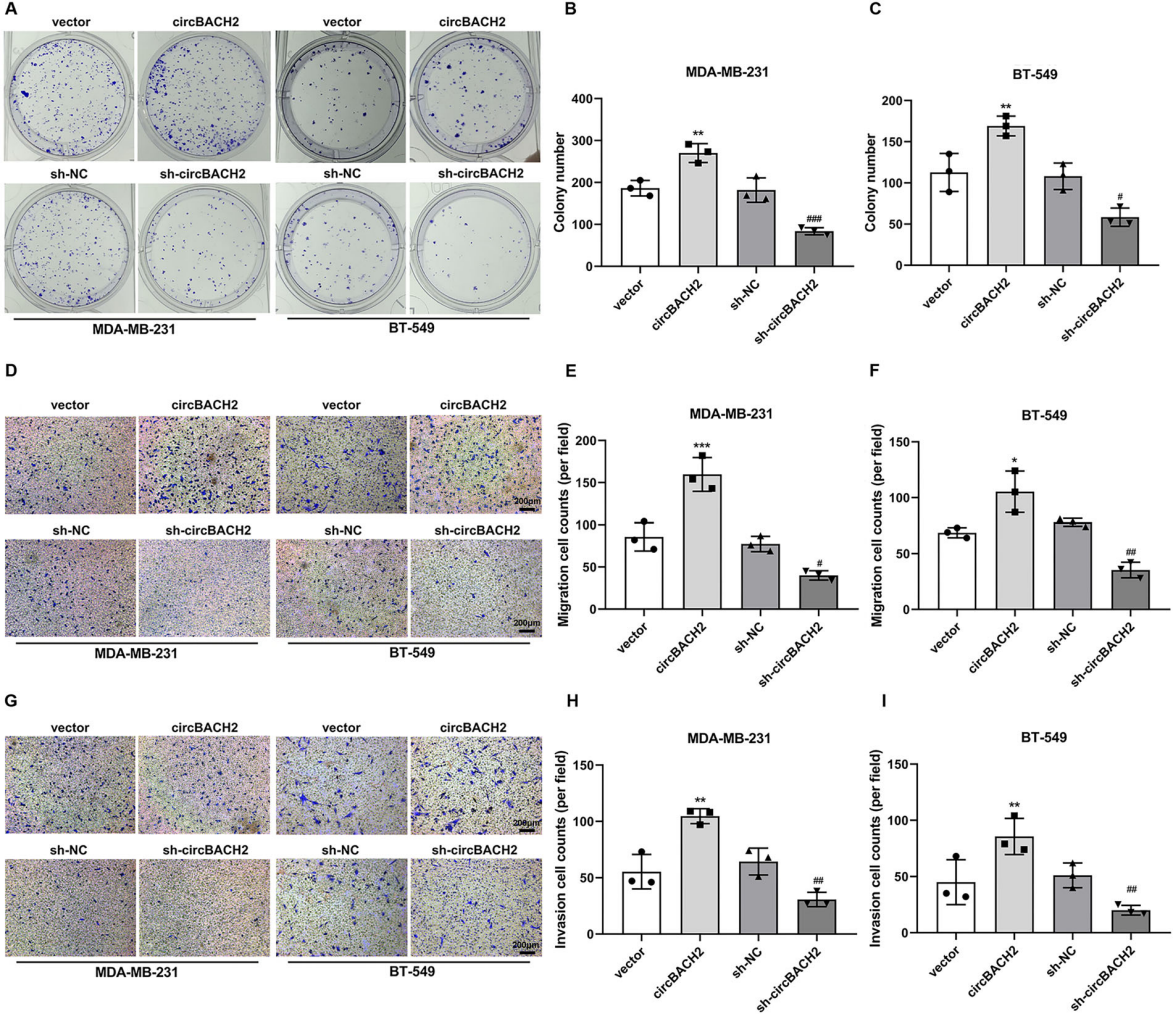
circBACH2 enhanced in vitro migration-invasiveness of TNBC cell lines. **A**–**C** The proliferation of MDA-MB-231 and BT-549 cells transfected with indicated vectors was evaluated using colony formation assay and quantified. **D**–**F** Migration of MDA-MB-231 and BT-549 cells transfected with indicated vectors was evaluated using Transwell assay and quantified. **G**–**I** Invasion of MDA-MB-231 and BT-549 cells transfected with indicated vectors was evaluated using Transwell assay and quantified. *P < 0.05, **P < 0.01, ***P < 0.001 vs vector; ^#^*P* < 0.05, ^##^*P* < 0.01, ^###^*P* < 0.001 vs sh-NC.

CircBACH2 was overexpressed in MCF-10A cells utilizing the transfection of the overexpression vector of circBACH2. As shown in Fig. 2A, in response to circBACH2 overexpression, the epithelial markers (E-cadherin and occludin^16^) protein levels were lessened, whereas mesenchymal marker (vimentin) and epithelial-mesenchymal transition (EMT) activators (slug, twist, and snail^17^) protein levels were increased. Meanwhile, compared with the mock vector, the circBACH2 overexpression vector declined the ratio of cells in the G0/G1 phase and boosted the proportion of cells in the S phase in MCF-10A cells (Fig. 2B, C). In the TNBC cell lines, circBACH2 was overexpressed or silenced utilizing the transfection of the overexpression vector of circBACH2 or sh-circBACH2 (Fig. 2D–G). Considering that sh-circBACH2#3 exhibited optimal inhibition efficiency in both MDA-MB-231 and BT-549 cells (Fig. 2F, G), we selected sh-circBACH2#3 (hereinafter referred to as sh-circBACH2) to silence circBACH2 in the following procedures. The results of Fig. 2H–K showed a distinct augment of the TNBC cells in S phase in response to circBACH2 overexpressing and an apparent decline of the TNBC cells in S phase in response to circBACH2 silencing. Following, as depicted in Fig. 3, colony formation, migration, and invasion were accelerated by circBACH2 overexpression and reduced by circBACH2 knockdown in the TNBC cell lines. Meanwhile, the transfection of circBACH2 overexpression vector significantly promoted the migration and invasion of MCF-10A cells (Supplemental Fig. 1).

### circBACH2 functioned as a sponge for miR-186-5p and miR-548c-3p in TNBC cell lines

**Fig. 4:**
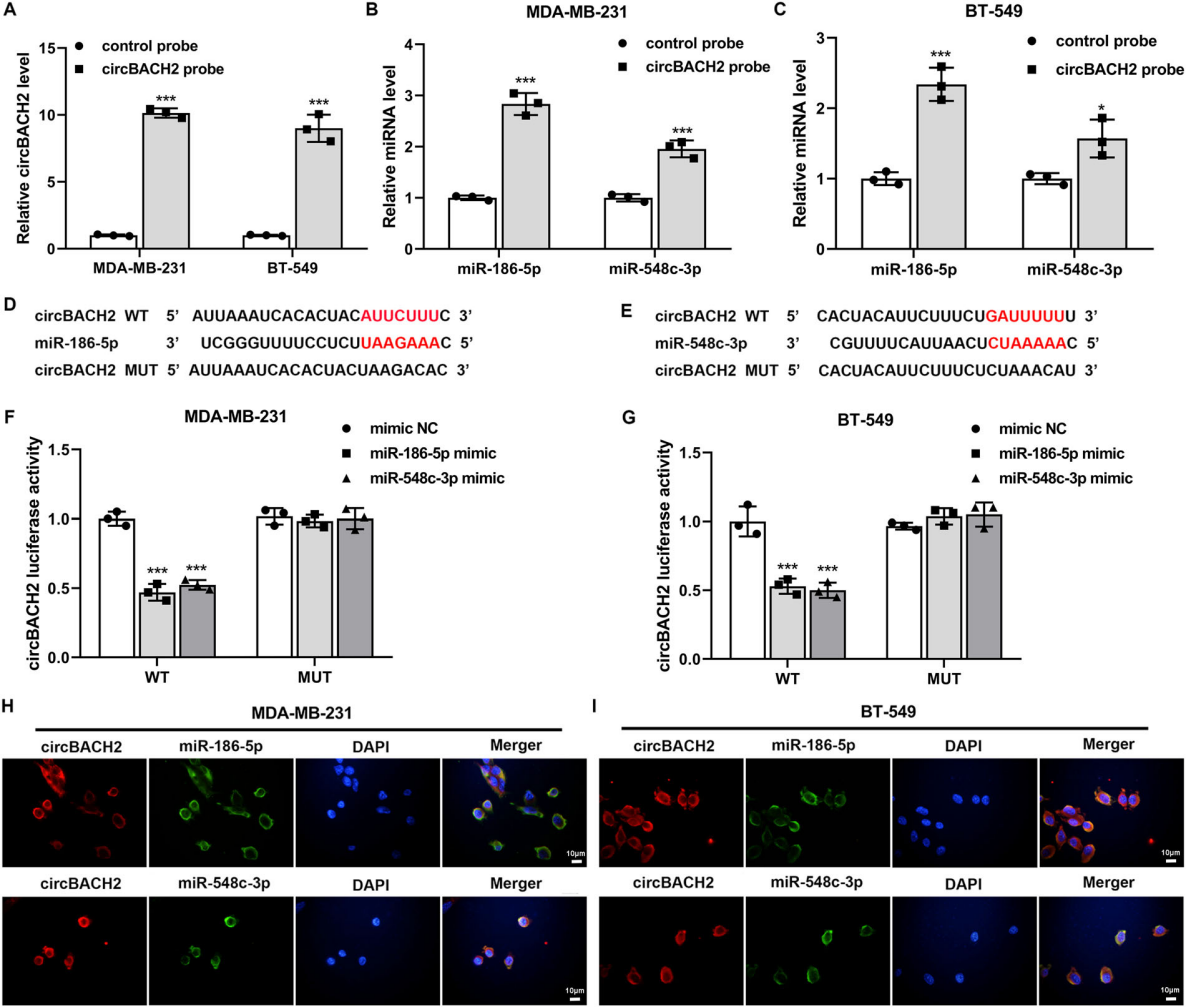
circBACH2 functioned as a sponge for miR-186-5p and miR-548c-3p in TNBC cell lines. **A**–**C** RNA pull-down assay was performed in MDA-MB-231 and BT-549 cells using a biotin-labeled circBACH2 probe, following by qRT-PCR. The control probe was used as a negative control. **D**, **E** The predicted binding sites between circBACH2 and miR-186-5p/miR-548c-3p. **F**, **G** The luciferase reporter assay was performed in MDA-MB-231 and BT-549 cells that were co-transfected with circBACH2 wild type (WT)/mutant type (MUT) luciferase vector and miR-186-5p mimic/miR-548c-3p mimic/mimic NC. **H**–**I** Fluorescence in situ hybridization (FISH) was executed to confirm the location of circBACH2 (red) and miR-186-5p/miR-548c-3p (green) in MDA-MB-231 and BT-549 cells. Nuclei were stained blue with DAPI. Scale bar = 10 µm. *P < 0.05, ***P < 0.001 vs control probe or mimic NC.

Subsequently, we explored whether circBACH2 exerted its tumorigenic action via the ceRNA mechanism in TNBC. Using circRNA Interactome (https://circinteractome.nia.nih.gov/), we determined two potential miRNAs, namely, miR-186-5p and miR-548c-3p, which had higher prediction scores for binding to circBACH2 and had been proven to be tumor suppressor genes in breast cancer^18,19^. First, the results of the RNA pull-down assay revealed that compared with the complex pulled down by the control probe, more abundant circBACH2, miR-186-5p, and miR-548c-3p were detected in the complex pulled down by the circBACH2 probe (Fig. 4A–C). Second, the TNBC cell lines were co-transfected with circBACH2 WT/MUT luciferase reporter (Fig. 4D, E) and miR-186-5p mimic/miR-548c-3p mimic/mimic NC. As shown in Fig. 4F, G, the luciferase activities of circBACH2 WT were prominently reduced in response to miR-186-5p/miR-548c-3p mimic transfection in both MDA-MB-231 and BT-549 cells, whereas the luciferase activities of circBACH2 MUT exhibited no obvious change. Finally, the FISH assay was executed to visually verify the co-localization of circBACH2 and miR-186-5p/miR-548c-3p in the cytoplasm of MDA-MB-231 and BT-549 cells (Fig. 4H, I).

### miR-186-5p and miR-548c-3p were downregulated in TNBC cancerous tissues

**Fig. 5:**
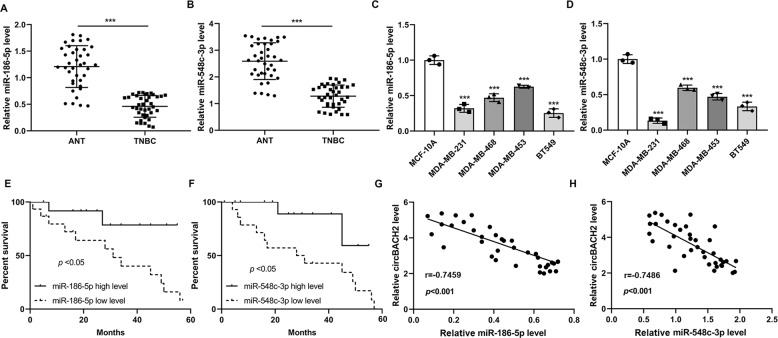
miR-186-5p and miR-548c-3p were downregulated in TNBC tissues. The expression levels of **A** miR-186-5p and **B** miR-548c-3p in TNBC (*n* = 38) and ANT tissues (*n* = 38). ***P < 0.001. The expression levels of **C** miR-186-5p and **D** miR-548c-3p in the normal mammary gland cell line MCF-10A and TNBC cell lines (MDA-MB-231, MDA-MB-468, MDA-MB-453, and BT-549). ***P < 0.001 vs MCF-10A. **E**, **F** The survival curve of patients with low/high miR-186-5p/miR-548c-3p level. **G**, **H** The correlation between circBACH2 level and miR-186-5p/miR-548c-3p level in TNBC tissues (*n* = 38).

Utilizing qRT-PCR, we determined the expression profiles of miR-186-5p and miR-548c-3p in TNBC tissues and TNBC cell lines. The results depicted that the expression levels of miR-186-5p and miR-548c-3p were obviously lessened in both TNBC cancerous tissues and TNBC cell lines (Fig. 5A–D). The TNBC patients with a lower miR-186-5p/miR-548c-3p level also exhibited a poor survival rate (Fig. 5E, F). Meanwhile, the circBACH2 level was negatively correlated with the miR-186-5p (*r* = −0.7459, *P* < 0.001)/miR-548c-3p (*r* = −0.7486, *P* < 0.001) level in TNBC cancerous tissues (Fig. 5G, H), which suggested that circBACH2 might function its role in TNBC through miR-186-5p and miR-548c-3p.

### CXCR4 was the downstream target of miR-186-5p and miR-548c-3p

**Fig. 6:**
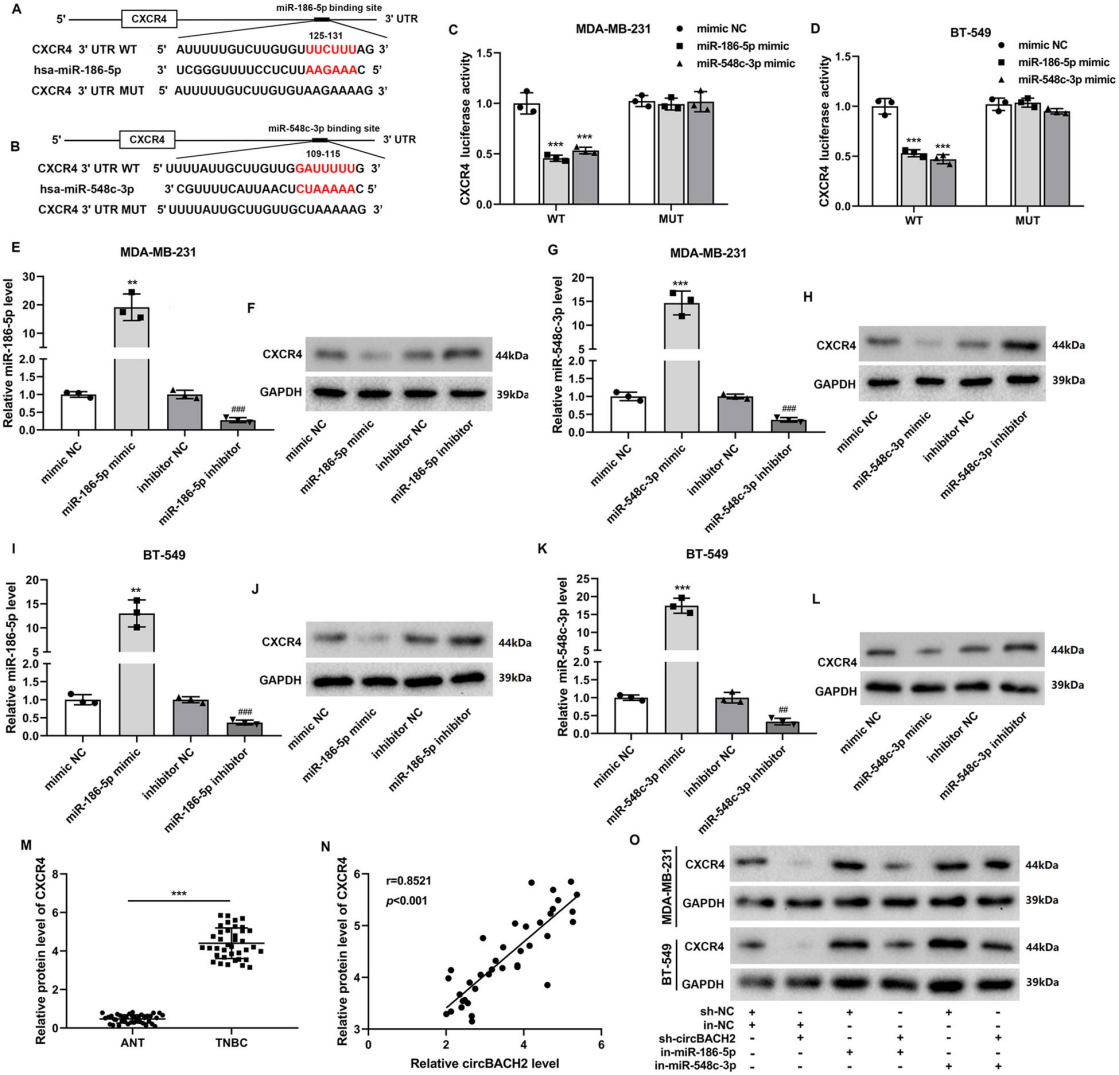
C-X-C chemokine receptor type 4 (CXCR4) was the downstream target of miR-186-5p and miR-548c-3p. **A**, **B** The predicted binding sites between miR-186-5p/miR-548c-3p and *CXCR4* mRNA. **C**, **D** The luciferase reporter assay was performed in MDA-MB-231 and BT-549 cells that were co-transfected with CXCR4 WT/MUT luciferase vector and miR-186-5p mimic/miR-548c-3p mimic/mimic NC. **E**–**L** MDA-MB-231 and BT-549 cells were transfected with miR-186-5p/miR-548c-3p mimic or miR-186-5p/miR-548c-3p inhibitor or corresponding negative controls (mimic NC or inhibitor NC). The expression levels of miR-186-5p/miR-548c-3p were measured by RT-PCR. The protein level of CXCR4 was measured by western blot. GAPDH was used as an internal control. **M** The protein level of CXCR4 was measured in TNBC cancerous tissues (*n* = 38) and ANT (*n* = 38). ***P < 0.001. **N** The correlation plot of circBACH2 level and CXCR4 protein level in TNBC cancerous tissues (*n* = 38). **O** MDA-MB-231 and BT-549 cells were divided into six groups: sh-NC + inhibitor NC (in-NC), in-NC + sh-circBACH2, sh-NC + in-miR-186-5p, sh-circBACH2 + in-miR-186-5p, sh-NC + in-miR-548c-3p, and sh-circBACH2 + in-miR-548c-3p. The protein level of CXCR4 was measured by western blot. GAPDH was used as an internal control. **P < 0.01, ***P < 0.001 vs mimic NC; ^##^*P* < 0.01, ^###^*P* < 0.001 vs inhibitor NC.

Using an online bioinformatics database TargetScan (http://www.targetscan.org/vert_72/), we found that CXCR4, a well-known promoter of TNBC growth and metastasis^20,21^, maybe the target gene of miR-186-5p and miR-548c-3p (Fig. 6A, B). The results shown in Fig. 6C, D show that the overexpression of miR-186-5p/miR-548c-3p markedly decreased the luciferase activity of CXCR4 WT but did not influence the luciferase activity of CXCR4 MUT in both MDA-MB-231 and BT-549 cells. Then, miR-186-5p/miR-548c-3p was overexpressed/knocked down in the TNBC cell lines utilizing transfections of the miR-186-5p mimic/miR-548c-3p mimic/miR-186-5p inhibitor/miR-548c-3p inhibitor (Fig. 6E, G, I, K). The results in Fig. 6F, H, J, L depicted that the CXCR4 protein level was decreased by miR-186-5p/miR-548c-3p overexpression, whereas raised by miR-186-5p/miR-548c-3p knockdown in the TNBC cell lines. Subsequently, we found that the protein level of CXCR4 in the TNBC cancerous tissues was significantly higher than that in ANT (Fig. 6M). Meanwhile, there was a positive correlation between the CXCR4 protein level and the circBACH2 expression level in the TNBC cancerous tissues (*r* = 0.8521, *P* < 0.001; Fig. 6N). In the TNBC cell lines, sh-circBACH2 apparently decreased CXCR4 protein level, which was then reversed by miR-186-5p/miR-548c-3p inhibitor (Fig. 6O).

### miR-186-5p and miR-548c-3p mediated the modulatory effect of circBACH2 on the proliferation, invasion, and migration of TNBC cell lines

**Fig. 7:**
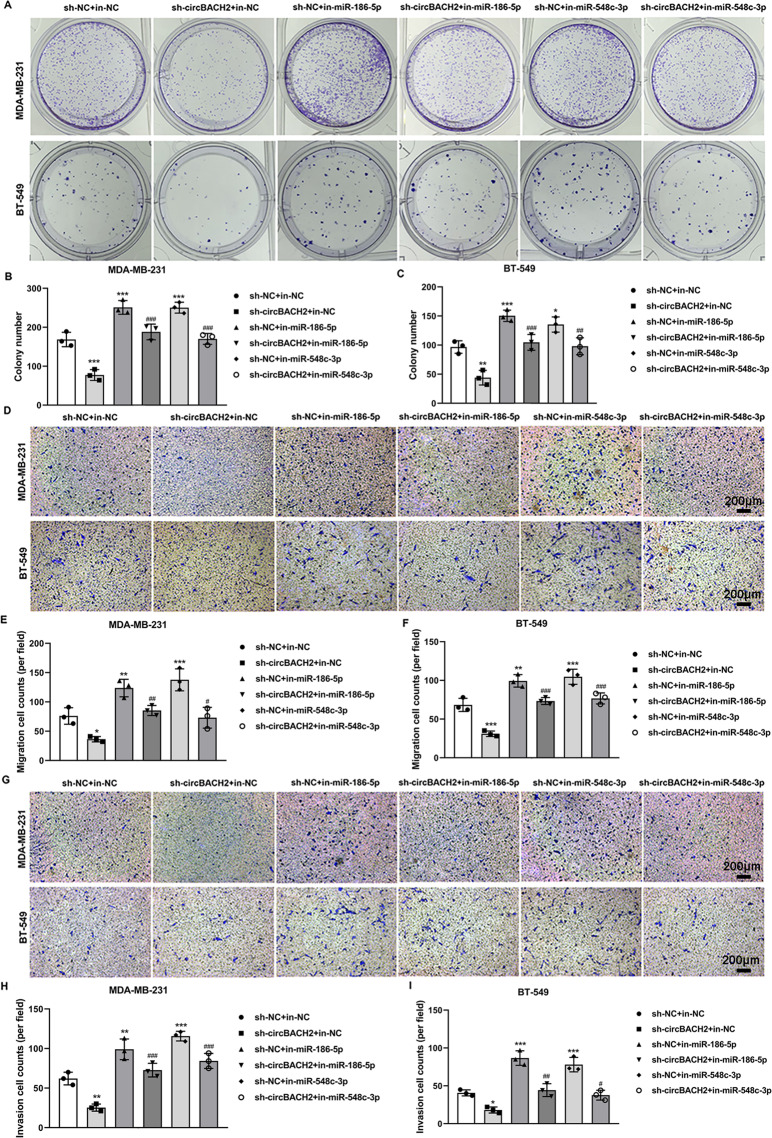
miR-186-5p and miR-548c-3p mediated the regulatory effect of circBACH2 on the progression of TNBC in vitro. MDA-MB-231 and BT-549 cells were divided into six groups: sh-NC + in-NC, in-NC + sh-circBACH2, sh-NC + in-miR-186-5p, sh-circBACH2 + in-miR-186-5p, sh-NC + in-miR-548c-3p, and sh-circBACH2 + in-miR-548c-3p. **A**–**C** Cell proliferation was evaluated using colony formation assay and quantified. **D**–**F** Cell migration was evaluated using Transwell assay and quantified. **G**–**I** Cell invasion was evaluated using Transwell assay and quantified. *P < 0.05, **P < 0.01, ***P < 0.001 vs sh-NC + in-NC; ^#^*P* < 0.05, ^##^*P* < 0.01, ^###^*P* < 0.001 vs in-NC + sh-circBACH2.

Afterward, we explored whether circBACH2 modulated the proliferation, migration, and invasion of TNBC cells through miR-186-5p and miR-548c-3p. As demonstrated in Fig. 7A–C, the silence of circBACH2 distinctly inhibited the colony formation in both MDA-MB-231 and BT-549 cells, while miR-186-5p/miR-548c-3p inhibitor promoted the colony formation in the presence of sh-circBACH2. Similarly, in response to the transfection of sh-circBACH2, the migration and invasion of the TNBC cell lines were suppressed (Fig. 7D–I). However, in the TNBC cell lines that were co-transfected with miR-186-5p/miR-548c-3p inhibitor + sh-circBACH2, miR-186-5p/miR-548c-3p inhibitor effectively promoted sh-circBACH2-suppressed cell migration and invasion (Fig. 7D–I). Considering that circBACH2 released the CXCR4 expression by sponging miR-186-5p and miR-548c-3p (Fig. 6O) and CXCR4 played a vital role in the progression of TNBC^20^, we concluded that circBACH2 promotes TNBC progression via the miR-186-5p/miR-548c-3p/CXCR4 axis.

### circBACH2 promoted the progression and metastasis of TNBC in vivo

**Fig. 8:**
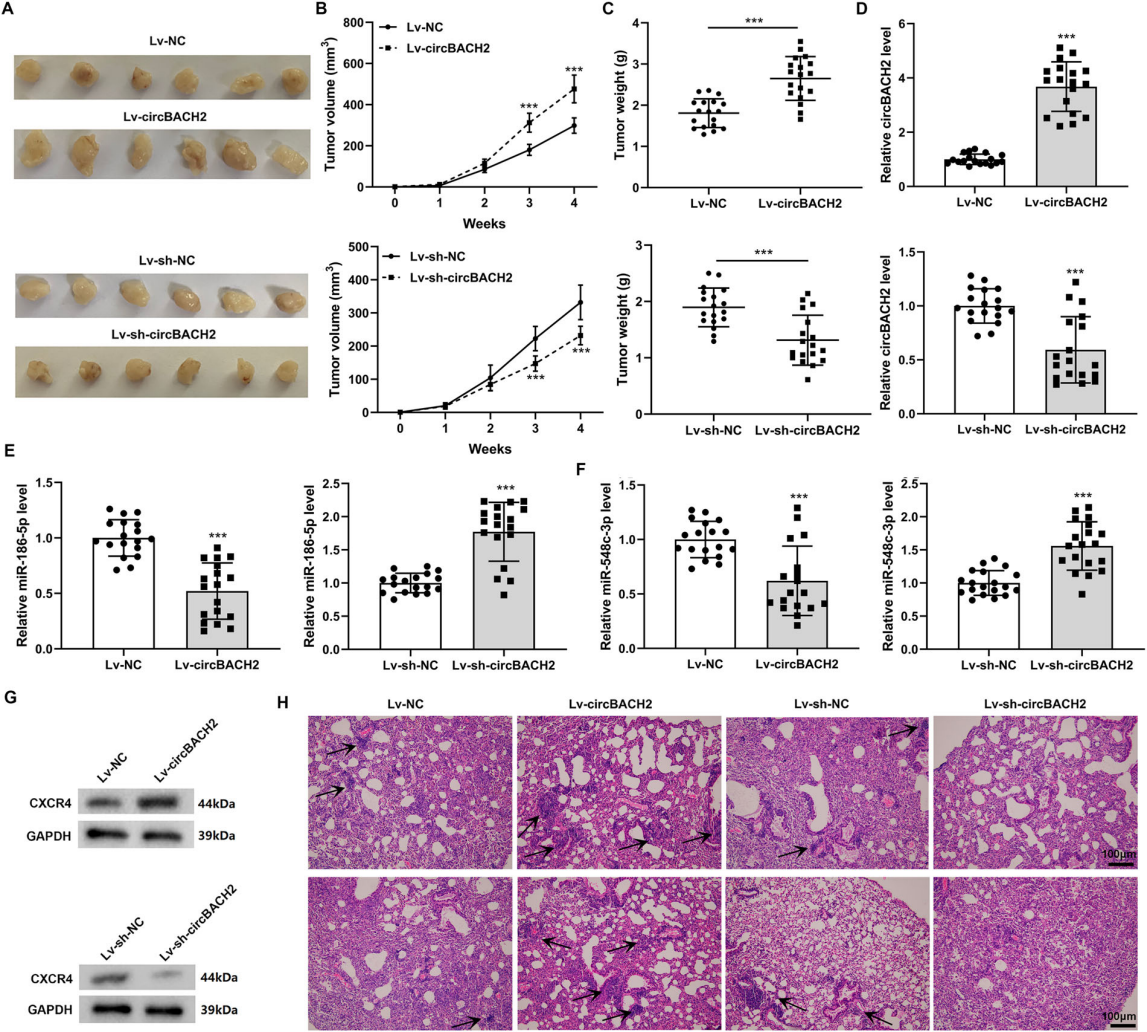
circBACH2 promoted the progression of TNBC in vivo. MDA-MB-231 cells were transfected with Lv-circBACH2 or Lv-sh-circBACH2 or corresponding controls (Lv-NC or Lv-sh-NC) and subcutaneously injected into the nude mice. **A** The xenografted tumors photos of mice (*n* = 6). The tumor **B** volumes and **C** weights of mice. ***P < 0.001. MDA-MB-231 cells were transfected with Lv-circBACH2 or Lv-sh-circBACH2 or corresponding controls (Lv-NC or Lv-sh-NC) and transplanted into the nude mice by tail vein injection. *n* = 6 of each group. **D** The expression level of circBACH2 in the lung tissues of mice. The expression levels of **E** miR-186-5p and **F** miR-548c-3p in the lung tissues of mice. **G** The CXCR4 protein level in the lung tissues of mice. **H** Representative HE staining (scale bar = 100 µm) for lung tissues of mice in each group. Arrows: metastatic nodules. ***P < 0.001 vs Lv-NC or Lv-sh-NC. **B**–**F** the experiment was repeated three times with tumor samples from six mice.

To verify the effect of circBACH2 on tumorigenesis in vivo, circBACH2-overexpressed, NC-overexpressed, circBACH2-knockdown, or NC-knockdown MDA-MB-231 cells, respectively, were subcutaneously injected into the mice. As demonstrated in Fig. 8A–C, the overexpression of circBACH2 remarkably raised the tumor volume and weight. On the contrary, the silence of circBACH2 lessened the tumor volume (Fig. 8A, B) and weight (Fig. 8C). To explore the effect of circBACH2 on the metastasis of TNBC in vivo, a lung metastasis model was established. Compared with mice transplanted with NC-overexpressed cells, the mice transplanted with circBACH2-overexpressed cells exhibited a more serious lung metastasis (Fig. 8D, H). However, the lung metastasis was almost entirely prevented in the mice transplanted with circBACH2-knockdown cells (Fig. 8D, H). Moreover, the expression levels of miR-186-5p and miR-548c-3p were lessened (Fig. 8E, F), whereas the CXCR4 protein level was raised (Fig. 8G) in the tumor tissues of the lung metastasis model mice that were transplanted with circBACH2-overexpressed cells. In the tumor tissues of the lung metastasis model mice transplanted with circBACH2-knockdown cells, the expression levels of miR-186-5p and miR-548c-3p were elevated (Fig. 8E, F), whereas the CXCR4 protein level was declined (Fig. 8G). These data suggest that the miR-186-5p/miR-548c-3p/CXCR4 axis took part in the modulatory effect of circBACH2 on the growth and metastasis of TNBC xenografts in nude mice, which was consistent with the data in vitro.

## Discussion

Recently, the effect of circRNAs on cancer progression has gained increasing attention. Several circRNAs have been determined as potential biomarkers for cancer^22–24^. However, the role of circRNAs in breast cancer, particularly in TNBC, remains unclear. In the current study, we discovered that circBACH2 was upregulated in TNBC cancerous tissues and its high expression showed a positive correlation with the malignant progression of TNBC patients. circBACH2 enhanced the expression of CXCR4 by adsorbing miR-186-5p and miR-548c-3p, thus promoting the proliferation and metastasis of TNBC cells. Finally, we confirmed that circBACH2 could slow down the growth and partly prevent the lung metastasis of TNBC in vivo, which indicating the potential of circBACH2 as a therapeutic target of TNBC.

CircBACH2 is derived from the BACH2 gene, a transcription factor that is responsible for the differentiation of innate and adaptive cellular lineages^25^. Previous studies have shown that BACH2 facilitates tumor growth by promoting tumor immunosuppression^26,27^. Moreover, hsa_circ_0001627, a circRNA derived from exon 2 of the BACH2 gene, has been proven to be highly expressed in papillary thyroid cancer (PTC) tissues and facilitates the malignant progression of PTC^28^. Consistent with previous research, we observed that the circBACH2 expression was enhanced in the TNBC patients and cell lines. Functionally, the interference of circBACH2 efficiently restrained TNBC progression and metastasis in vitro and in vivo. Moreover, by analyzing the correlation between circBACH2 expression level and the clinical-pathological features of TNBC patients, we found that circBACH2 may be a promising biomarker for the malignant progression of TNBC. Considering the above findings, we identified the function and clinical significance of circBACH2 in TNBC for the first time. As reported in the latest research, methyltransferase-like 3 is downregulated in TNBC tissues and its low expression is associated with the terrible prognosis of TNBC patients^29^. Considering that *N*6-methyladenosine (m6A) modification is the most abundant modification on eukaryotic RNA molecules and regulates the degradation and splicing of target RNAs, including circRNAs^30^. We suspected that the elevation of the circBACH2 level during TNBC may be related to the m6A modification.

Increasing evidence points out that circRNAs modulate the development of cancer by acting as sponges of miRNAs. For instance, circRNA_101996 adsorbed miR-8075 by “sponge action”, and then enhanced the expression of the targeting protein Xenopus kinesin-like protein 2, thereby facilitating cervical cancer proliferation^31^. In breast cancer, circTADA2As depressed cell migration by modulating the miR-203a-3p/suppressor of the cytokine signaling 3 axis^15^. In the present study, we revealed the function of circBACH2 as the sponge for miR-186-5p and miR-548c-3p. The reduced expression of miR-186-5p has been observed in breast cancer and the overexpression of miR-186-5p restrained the EMT process of breast cancer cells^18^. Similarly, the antitumor effect of miR-548c-3p in breast cancer has been verified in the research of Guo et al.^19^. In the present study, we found that the interference of miR-186-5p/miR-548c-3p evidently accelerated the colony formation, migration, and invasion of MDA-MB-231 and BT-549 cells, proving that miR-186-5p and miR-548c-3p also possess antitumor effects in TNBC.

CXCR4 is a highly conservative receptor that plays a key role in several cancer types^32^. The combination of CXCR4 and its ligand stromal cell-derived factor-1 could activate the phosphoinositide 3-kinase/protein kinase B signaling pathways^33^, which are involved in the modulation of the proliferation and survival of cancer cells^34^. Compared with other subtypes of breast cancer, CXCR4 expression was specifically raised in the TNBC patients; the high expression level of CXCR4 predicted a high malignant grade and a high possibility of recurrence^35^. Accumulating evidence has validated that CXCR4 inhibitors, such as the CXCR4 protein antagonist and the CXCR4-targeted nanocarriers, could efficaciously repress the growth and metastasis of TNBC cells^36,37^, proving that CXCR4 is a promising target for the clinical treatment of TNBC. Herein, we defined CXCR4 as the target gene of miR-186-5p and miR-548c-3p. Furthermore, the interference of miR-186-5p/miR-548c-3p abrogated the inhibitory effect of sh-circBACH2 on proliferation, invasion, migration, and CXCR4 expression in the TNBC cells, suggesting that the promoting effect of circBACH2 on TNBC progression and metastasis was dependent on CXCR4.

Overall, the current study found that circBACH2 adsorbed miR-186-5p and miR-548c-3p to regulate CXCR4 expression, thus accelerating the progression and metastasis of TNBC. We defined circBACH2 as an oncogenic circRNA in TNBC and revealed its clinical significance for TNBC patients, hoping to provide a novel therapeutic target for TNBC.

## Supplementary information

Supplemental

Supplemental Figure 1
