# Supplementary material for: Up-regulated circBACH2 contributes to cell proliferation, invasion, and migration of triple-negative breast cancer
Source: Cell Death Dis. 2021 Apr 19;12(5):412. doi: 10.1038/s41419-021-03684-x (PMC8055688; doi:10.1038/s41419-021-03684-x)
Supplement: Supplementary file 1 — Supplemental [file 41419_2021_3684_MOESM1_ESM.docx]

**Supplemental Figure 1** (A) MCF-10A cells were transfected with circBACH2 or vector. (B-D) The migration and invasion of MCF-10A cells transfected with circBACH2 or vector were measured.
