# Supplementary figures and images for: Up-regulated circBACH2 contributes to cell proliferation, invasion, and migration of triple-negative breast cancer
Source: Cell Death Dis. 2021 Apr 19;12(5):412. doi: 10.1038/s41419-021-03684-x (PMC8055688; doi:10.1038/s41419-021-03684-x)

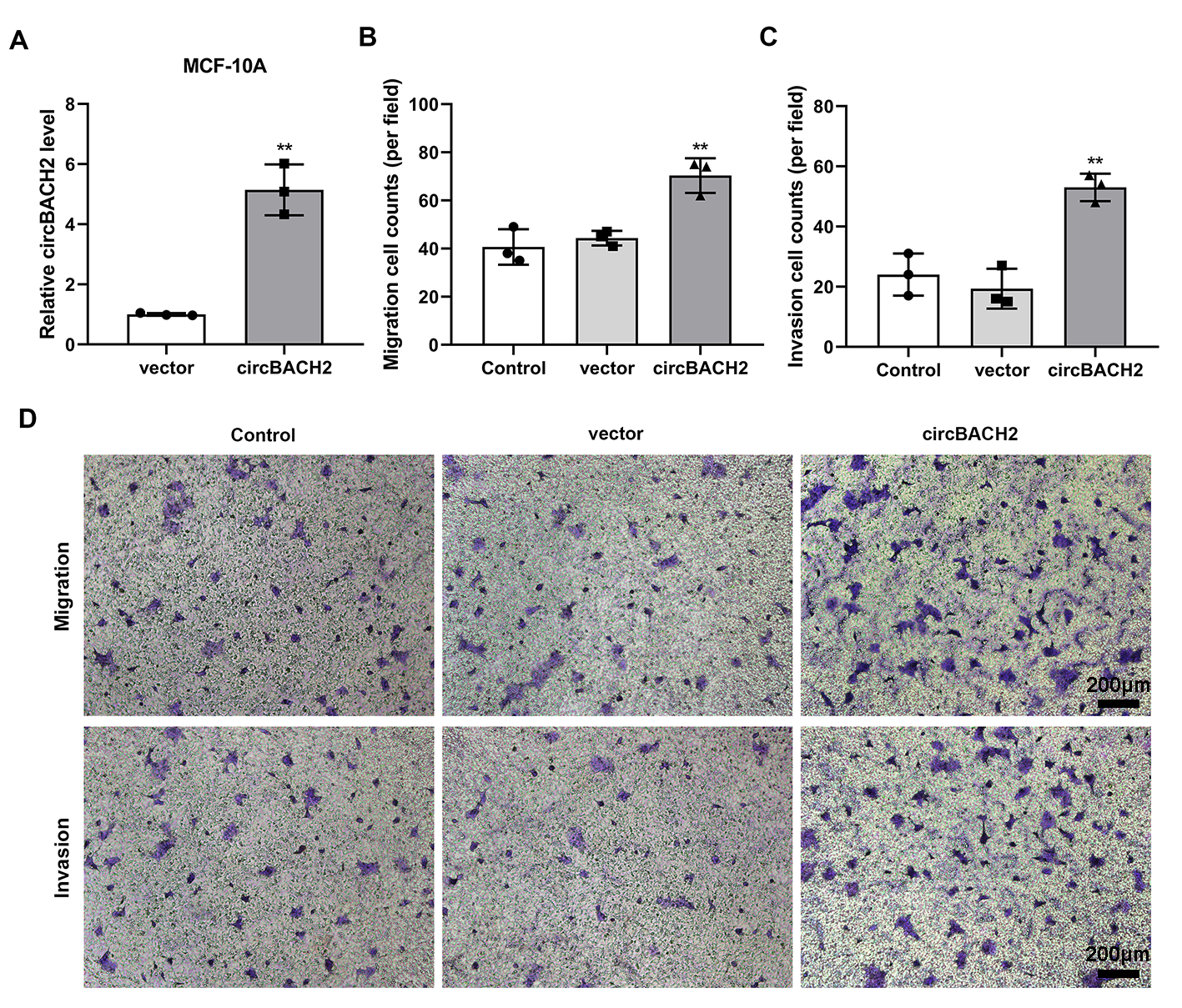

Supplement: Supplementary file 2 — Supplemental Figure 1 [file 41419_2021_3684_MOESM2_ESM.tif]
